# Inhibition of VEGFR-3 by SAR131675 decreases renal inflammation and lymphangiogenesis in the murine lupus nephritis model

**DOI:** 10.1038/s41420-025-02624-4

**Published:** 2025-07-12

**Authors:** Tian Wang, Wenjia Li, Ji-Hyun Yeom, Zhiheng Liu, Kyoung Min Kim, Kyung Pyo Kang

**Affiliations:** 1 Department of Internal Medicine, Research Institute of Clinical Medicine, Jeonbuk National University Medical School, Jeonju, Republic of Korea; 2 Central Hospital Affiliated to Shandong First Medical University, Jinan, Shandong People’s Republic of China; 3 Medical Integration and Practice Center, Shandong University, Jinan, Shandong People’s Republic of China; 4 Biomedical Research Institute, Jeonbuk National University Hospital, Jeonju, Republic of Korea; 5 Department of Pathology, Jeonbuk National University Medical School, Jeonju, Republic of Korea

**Keywords:** Lupus nephritis, Immunopathogenesis

## Abstract

Systemic lupus erythematosus (SLE) is a chronic autoimmune disease characterized by immune-complex deposits and inflammatory cell infiltrations in multiple organs. Approximately half of lupus patients have nephritis. Lymphangiogenesis is the proliferation of lymphatic vessels (LVs), which regulate tissue fluid homeostasis and immune cell trafficking, responding to the tissue environment. In this study, we evaluated the therapeutic effect of SAR131675, a selective VEGFR-3 inhibitor, on the murine lupus nephritis model by regulating inflammation and lymphangiogenesis. We evaluated biopsy-proven lupus nephritis with immunohistochemical staining for D2-40, a marker for human lymphatic endothelial cells. For animal experiments, 7- to 8-week-old male BALB/c mice were used. For the induction of a lupus-like model, the dorsal skin of mice was shaved and given topical treatment every other day with 100 μg resiquimod dissolved in 100 μL acetone during the 8-week treatment. We had renal histology and immunofluorescent study for inflammatory cells and lymphatic vessels. We also had a qRT-PCR and Western blot analysis to evaluate inflammatory cytokines and chemokines, lymphangiogenic factors, and TLR7/type I IFN response. A human study found that the higher the revised ISN/RPS LN histopathological classification and modified NIH activity indexes, the more D2-40 (+) lymphatic vessels were expressed in the tubulointerstitial areas. Inhibition of VEGFR-3 by oral SAR131675 treatment decreased the resiquimod-induced glomerular and tubulointerstitial inflammation and attenuated LYVE-1 (+) lymphatic vessel expression in the murine lupus model. Treatment SAR131675 decreased the resiquimod-induced increase of proinflammatory cytokines and chemokines by regulating TLR7/MyD88/IFN-α expression. This study suggests the therapeutic potential of targeting lymphatic proliferation by VEGFR-3 inhibition in lupus nephritis. Modulation of the lymphatic network may provide a novel approach to treating chronic inflammation and attenuating renal autoimmune response.

## Introduction

Systemic lupus erythematosus (SLE) is characterized by loss of self-tolerance against nuclear autoantigens, aberrant autoimmune response, and multi-organ inflammation [1–3]. Various factors, including genetic, environmental, hormonal, epigenetic, and immunoregulatory factors, act sequentially or simultaneously on our immune system [2]. According to the population-based study, the prevalence of lupus nephritis (LN) steadily increased [4]. More importantly, the mortality of LN patients was six times higher than that of the general population, and 13% of patients developed end-stage renal disease (ESRD), requiring renal replacement therapy [4]. Therefore, increasing disease burden and poor long-term outcomes emphasize the need to develop new therapeutic strategies for treating patients with LN.

Lymphatic systems regulate tissue fluid homeostasis, immune surveillance, and dietary fat absorption [5]. Lymphangiogenesis, proliferating preexisting lymphatic vessels, occurs under certain pathological conditions, such as tissue inflammation, wound healing, and tumor metastasis [5, 6]. Lymphatic vessels also contribute to recognizing the inflammatory reactions in allograft rejection by forming tertiary lymphoid organs (TLOs) [7, 8]. Inhibition of vascular endothelial growth factor (VEGF)-C/D and vascular endothelial growth factor receptor 3 (VEGFR-3) suggests a lymphatic vessel-targeted immunomodulatory therapy in cardiac allograft rejection and arteriosclerosis model [9]. Therefore, targeting lymphatic vessels to regulate inflammation suggests a novel therapeutic insight for treating chronic inflammation, including autoimmune disease, chronic graft rejection, persistent infection, and cancer [10].

TLOs are accumulations of lymphoid cells in non-lymphoid tissue with chronic inflammation resembling secondary lymphoid organs (SLOs) such as lymph nodes, spleen, tonsils, and specific tissues in various mucous membrane layers [10–12]. TLOs are usually present in chronic inflammation in processes such as autoimmune disease [13], chronic allograft rejection [14], and tumors like ovarian cancer, non-small cell lung cancer, colorectal cancer, breast, stomach, liver cancers, and melanoma [15]. TLOs are characterized by similar structures to SLOs, with cellular, chemokine, and vascular components, including T and B cell compartments, antigen-presenting cells like follicular dendritic cells and stromal cells, high endothelial venules, and lymphatic vessels [10]. Therefore, elucidating the regulatory mechanisms of TLOs contributes to developing novel therapeutic strategies in autoimmune disease and cancer-associated inflammatory processes.

In LN, active glomerular lesions are associated with results from a systemic break in B cell tolerance [16] and characterized by immune complex deposition in the subendothelial and mesangial lesions, which contain cationic anti-deoxyribonucleic acid (DNA) antibodies and antibodies against the collagen-like region of C1q [2]. Tubulointerstitial and vascular lesions are also important determinants for the prognosis of LN [17–19]. Local lymphoid aggregates with T and B cells have been observed within the kidney in patients with LN [20]. The exact roles of this lymphoid aggregate remain to be elucidated. Our preliminary work established the resiquimod-induced LN model, which shows severe glomerular and tubulointerstitial lesions with lymphoid aggregation around small arteries. We would like to know the roles of these lymphoid aggregations in the LN model and their clinical relevance. Therefore, we investigated the relationship between lymphatic vessel expression and the revised International Society of Nephrology/Renal Pathology Society (ISN/RPS) histopathologic classification of LN and the modified National Institutes of Health (NIH) activity indexes in human LN and the effect of VEGFR-3 inhibition on lymphoid aggregations and lymphatic vessel expression in the resiquimod-induced LN model.

## Results

### Association of lymphatic vessel expression with histopathological activity and serologic markers in Lupus Nephritis

**Fig. 1:**
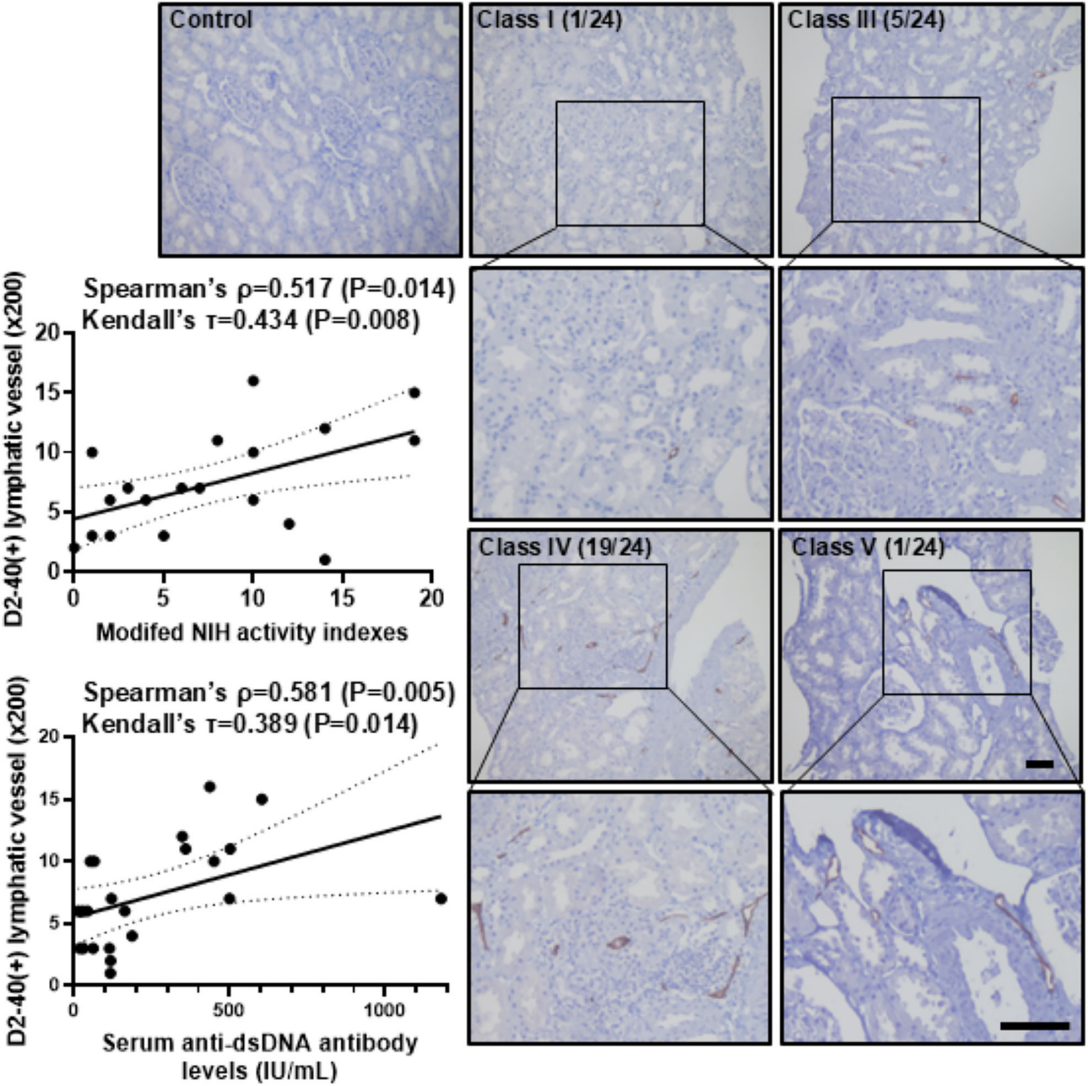
The correlation between the expression of D2-40 (+) lymphatic vessels and modified NIH activity indexes and serum anti-dsDNA antibody levels. Representative sections of human kidney tissues that were pathologically diagnosed with LN (class I to V, *n* = 22) were immunohistochemically stained with D2-40, a human lymphatic vessel marker, with magnification of 100× and 200×. Scale bar = 100 μm. D2-40(+) lymphatic vessels were counted in magnification of 200×. Each section shows the lupus class I, III, IV, and V with modified NIH activity indexes. Significant correlations were observed between the modified NIH activity indexes of LN and D2-40 (+) lymphatic vessel expression (Spearman’s *ρ* = 0.517, *P* = 0.014; Kendall’s τ = 0.434, *P* = 0.008), as well as serum anti-dsDNA antibody levels and D2-40 (+) lymphatic vessel expression (Spearman’s *ρ* = 0.581, P = 0.005; Kendall’s τ = 0.389, *P* = 0.014). LN, lupus nephritis.

To determine whether lymphatic vessel expression would correlate with the revised ISN/RPS histopathologic classification of LN [21], we enrolled 22 biopsy-proven LN patients. The kidney sections were immunohistochemically stained for anti-D2-40 antibody that detected a fixation-resistant epitope on a 40 kDd O-linked sialoglycoprotein expressed in human lymphatic endothelial cells, not in the blood vessels [22, 23]. Active lesions in this ISN/RPS classification are generally higher inflammatory or proliferative glomerular and tubulointerstitial lesions [24, 25]. Table 1 presents their clinical characteristics, including renal function and urine protein excretion. There was no significant difference in clinical characteristics between each LN class. However, D2-40 (+) lymphatic vessel expression per high-power field (HPF) significantly increased with LN class III to IV, with increased the modified NIH activity indexes (Spearman’s *ρ* = 0.517, *P* = 0.014; Kendall’s τ = 0.434, *P* = 0.008) (Fig. 1). We also evaluated the relationship between clinical parameters such as serum anti-double-stranded deoxyribonucleic acid (dsDNA) antibody levels, serum complement levels, urine albumin excretion, and D2-40 (+) lymphatic vessel expression. They are only correlated with serum anti-dsDNA antibody level and D2-40 (+) lymphatic vessel expression per HPF (Spearman’s *ρ* = 0.581, *P* = 0.005; Kendall’s τ = 0.389, *P* = 0.014) (Fig. 1). These results suggest that the revised ISN/RPS LN histopathologic classification and the modified NIH activity indexes, and serum anti-dsDNA antibody levels are correlated with the number of lymphatic vessel expressions in human LN.

**Table 1:** Clinical characteristics of patients with biopsy-proven lupus nephritis.

LN class	I	II	III	IV	V	IV + V
Age (mean ± SD)	18	67	31 (±14)	26.7 (±12.3)	40.7 (±12.2)	24
n(M:F)	1 (0:1)	1 (0:1)	3 (0:3)	10 (1:9)	6 (2:4)	1 (0:1)
WBC (/mm^3^)	3670	4020	7670 (±4146)	9265 (±5196)	8295 (±2642)	3290
Hgb (g/dL)	9.4	9.7	10.1 (±1.4)	10.6 (±1.8)	12.3 (±1.7)	7.9
PLT (/mm^3^)	112,000	131,000	180,000 (±7640)	247,000 (±110,000)	226,000 (±91,800)	213,000
ESR (mm/h)	94	87	11 (±1.7)	15.5 (±12.4)	38.5 ± (38.5)	27
Creatinine (mg/dL)	0.38	1.0	0.56 (±0.1)	0.89 (±0.45)	0.56 (±0.08)	0.7
eGFR (ml/min/1.73m^2^)	155	58.4	129 (±13.8)	95.8 (±34.7)	122 (±17.6)	122
CRP (mg/L)	4.4	2.7	1.7 (±2.2)	1.8 (±3.4)	2.1 (±4.4)	1.5
C3 (mg/dL)	79	45.4	30.7 (±7.4)	50.3 (±11.9)	66.7 (±21.3)	65.9
C4 (mg/dL)	8.3	2.5	2.3 (±1.4)	6.5 (±4.1)	15.3 (±12.1)	7.7
Anti-dsDNA (IU/mL)	118	43	140 (±40.6)	466 (±293)	36 (±22.1)	52
uPCR (mg/g Cr)	9581	6615	1442 (±616)	4584 (±2995)	4635 (±3459)	2400

*anti-dsDNA* anti-double-stranded deoxyribonucleic acid (reference range: <15 IU/mL), *C3* complement component 3 (reference range: 80~160 mg/dL), *C4* complement component 4 (reference range: 16~48 mg/dL), *eGFR* estimated glomerular filtration rate (calculated by CKD-EPI equation), *CRP* C-reactive protein (reference range 0~5.0 mg/L), *uPCR* urine protein/creatinine ratio, *Hgb* hemoglobin, *PLT* platelet

### Inhibition of VEGFR-3 decreases resiquimod-induced lupus nephritis

**Fig. 2:**
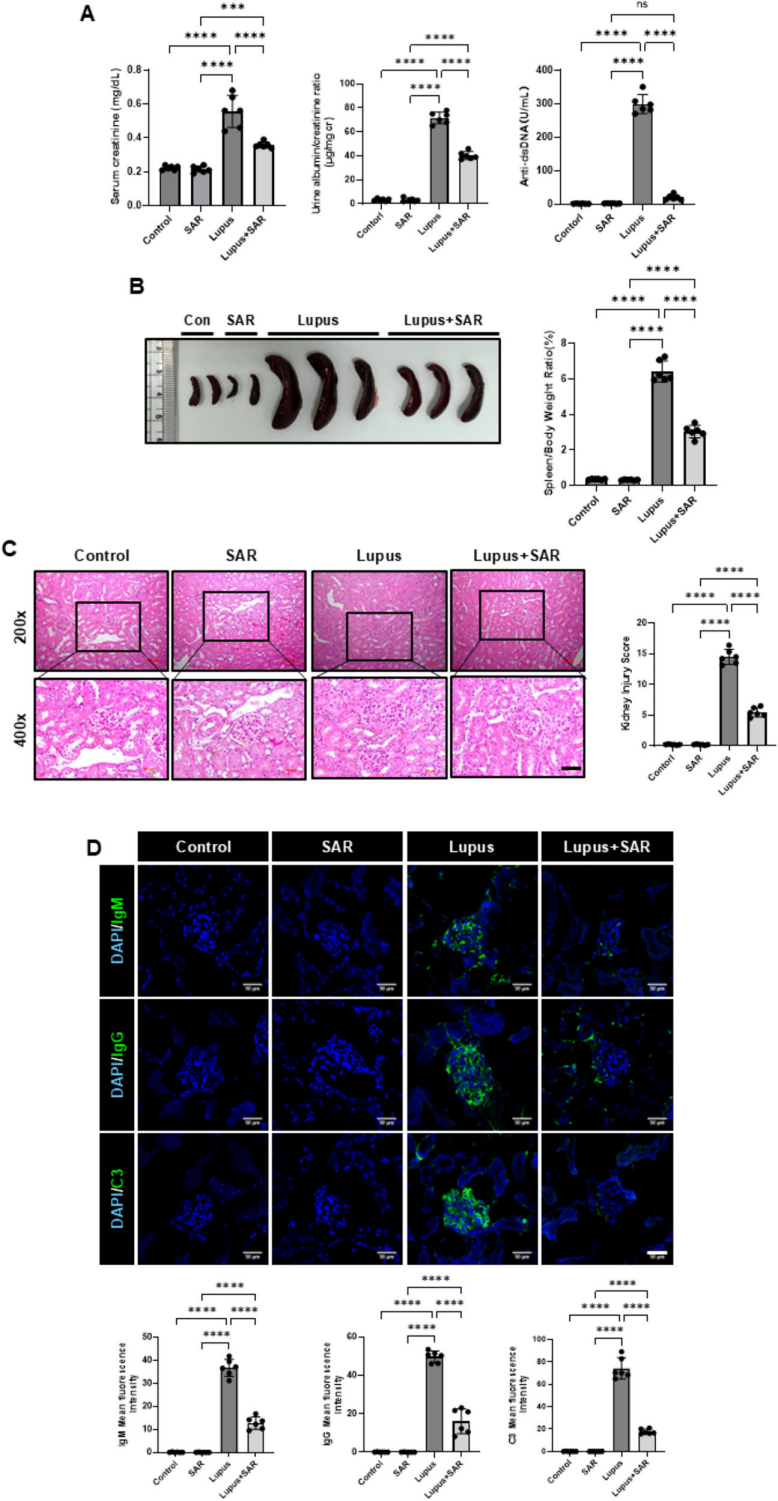
Inhibition of VEGFR-3 by SAR131675 decreases the resiquimod-induced lupus nephritis. **A** Serum creatinine levels, urine albumin/creatinine ratio, and anti-dsDNA antibody levels after SAR131675 treatment in the resiquimod-induced LN. **B** Gross finding of the spleen after SAR131675 treatment in the resiquimod-induced LN. The bar graph shows the spleen/body weight ratio (%). **C** Representative sections of kidneys from each experimental group of SAR131675 treatment in the resiquimod-induced LN were stained with Hematoxylin and Eosin (H&E) with magnification of 200× and 400×. Scale bar = 50 μm. The bar graph shows semi-quantitative scoring of kidney injury by H&E in each experimental group. **D** Representative sections of kidneys from each experimental group of SAR161675 treatment in the resiquimod-induced LN were immunofluorescence stained with glomerular immune complex IgM, IgG, and C3; Scale bar = 50 μm. The bar graph shows a mean fluorescence intensity in each experimental group. Statistical significance was determined using one-way ANOVA followed by Tukey’s post-hoc test. Data are expressed as mean ± SD. ****P < 0.0001 versus each group. Control, vehicle-treated group; SAR, SAR131675-treated group; Lupus, resiquimod-treated group; Lupus+SAR, resiquimod and concomitantly SAR131675-treated group. Anti-dsDNA antibody, anti-double-stranded DNA antibody, C3 complement component 3, DAPI 4’,6-diamidino-2-phenylindole, IgM immunoglobulin M, IgG immunoglobulin g, LN lupus nephritis.

Next, we evaluated whether inhibiting lymphatic vessel expression by SAR131675, a selective VEGFR-3 inhibitor, might benefit LN. First, we established a lupus-like model through the topical treatment of resiquimod in Balb/c mice. Eight weeks of topical treatment with resiquimod increased serum creatinine, urine albumin excretion, and serum anti-dsDNA levels, suggesting that we could successfully induce the resiquimod-induced LN model. SAR131675 treatment decreased the resiquimod-induced increase of serum creatinine, urine albumin excretion, and serum anti-dsDNA levels in the LN model (Fig. 2A). Figure 2B shows gross findings of the spleen after topical treatment with resiquimod. After topical treatment with resiquimod, the spleen/body weight ratio significantly increased; however, SAR131675 treatment decreased resiquimod-induced splenomegaly.

Histologically, eight weeks of topical treatment of resiquimod had induced glomerular inflammation, mesangial cell proliferation, crescent formation, or necrotic changes with severe tubulointerstitial inflammatory cell infiltration compared with vehicle or SAR131675-only treated groups. However, in the LN model, SAR131675 treatment significantly reduced the resiquimod-induced increase in the kidney injury score (Fig. 2C). LN is characterized by glomerulonephritis with immune complex deposition and the characteristic “full house” pattern on immunofluorescence staining [24, 26]. We evaluated glomerular IgM, IgG, and C3 deposition by immunofluorescence staining to demonstrate the immune mechanism of resiquimod-induced LN. The lupus model showed significantly increased glomerular IgM, IgG, and C3 expressions. However, the SAR131675 treatment ameliorated resiquimod-induced glomerular deposition of IgM, IgG, and C3 (Fig. 2D). The data suggest that the inhibition of VEGFR-3 by SAR131675 decreases resiquimod-induced LN functionally and histologically.

### Inhibition of VEGFR-3 decreases LYVE-1 (+) lymphatic vessel expression in the resiquimod-induced lupus nephritis

**Fig. 3:**
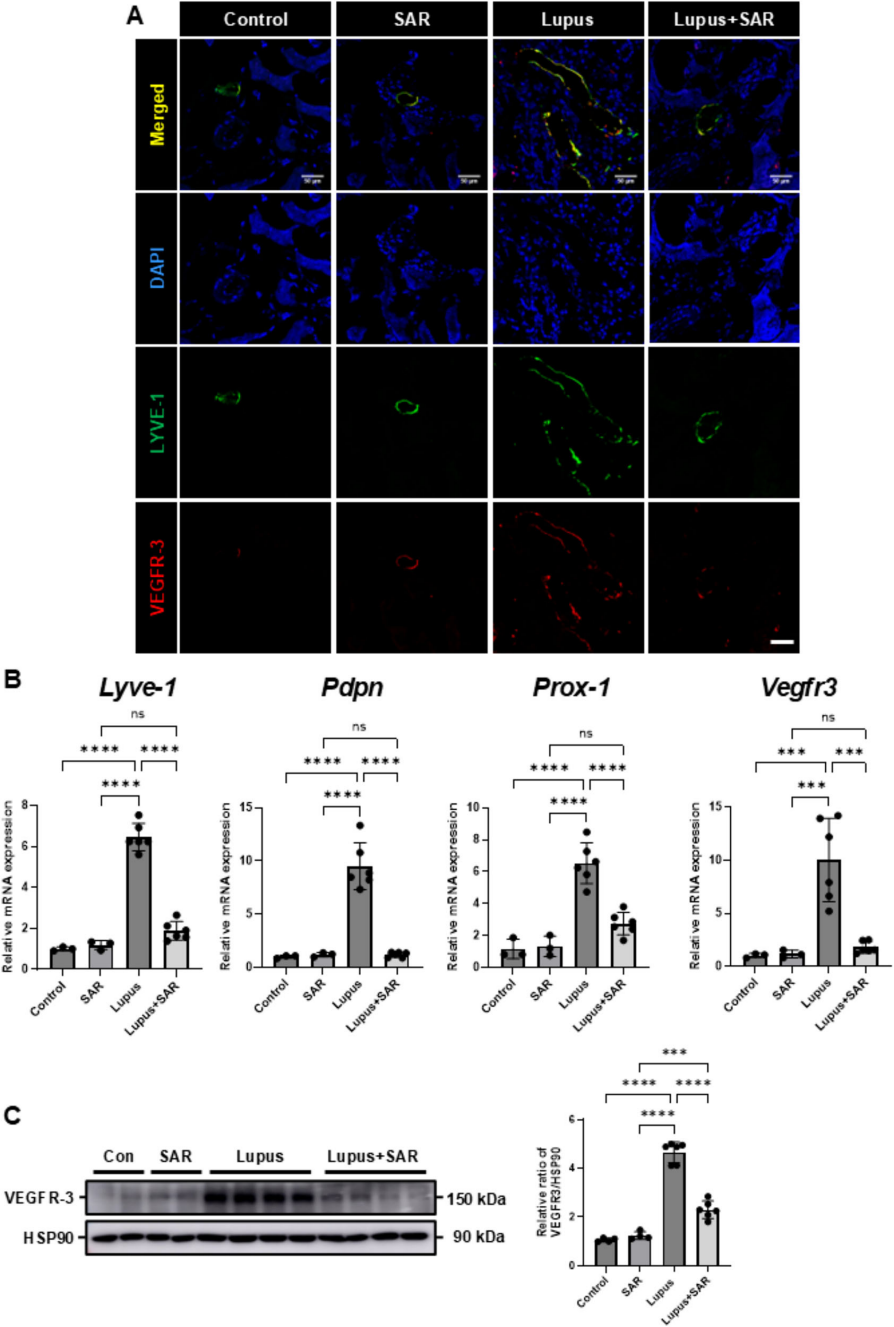
Treatment of SAR131675 decreases LYVE-1 (+) lymphatic vessel expression in the resiquimod-induced lupus nephritis. **A** Representative sections of kidneys from each experimental group of SAR131675 treatment in the resiquimod-induced LN were immunofluorescence stained with LYVE-1 and VEGFR-3; Scale bar=50 μm. **B** Quantitative RT-PCR analyses for *Lyve-1, Pdpn, Prox-1*, and *Vegfr-3* mRNA expression were performed using mRNA from each experimental group of SAR131675 treatment in the resiquimod-induced LN. Expression levels are normalized to *Gapdh*. **C** Representative Western blot analysis of VEGFR-3 protein expression from each experimental group of SAR131675 treatment in the resiquimod-induced LN. The graph shows the densitometric quantification of Western blot bands by ImageJ software. Expression levels are normalized to HSP90. Statistical significance was determined using one-way ANOVA followed by Tukey’s post-hoc test. Data are expressed as mean ± SD. ***P < 0.001, ****P < 0.0001 versus each group. Control, vehicle-treated group; SAR, SAR131675-treated group; Lupus, resiquimod-treated group; Lupus+SAR, resiquimod and concomitantly SAR131675-treated group. DAPI 4’,6-diamidino-2-phenylindole, LN lupus nephritis, LYVE-1 lymphatic vessel endothelial hyaluronan receptor-1, Pdpn podoplanin, Prox-1 prospero-related homeobox 1, VEGFR-3 vascular endothelial growth factor receptor-3.

We examined lymphatic vessel expression following VEGFR-3 inhibition through SAR131675 treatment. This study utilized two lymphatic endothelial cell markers, LYVE-1 (lymphatic vessel endothelial hyaluronan receptor-1) and VEGFR-3, as LYVE-1 is also expressed in blood vessels and macrophages [27, 28]. The resiquimod-induced LN significantly increased the expression of LYVE-1(+) lymphatic vessels. They were also co-expressed in VEGFR-3 with LYVE-1(+) lymphatic vessels in resiquimod-induced LN. After treatment with SAR131675, a selective VEGFR-3 inhibitor, LYVE-1(+) lymphatic vessel expression was significantly decreased in resiquimod-induced LN (Fig. 3A). We assessed the mRNA expression of lymphatic vessel markers, including *Lyve-1*, podoplanin (*Pdpn*), Prospero Homeobox-1 (*Prox-1*), and *Vegfr3*. Induction of LN with resiquimod resulted in a notable increase in the mRNA levels of *Lyve-1*, *Pdpn*, *Prox-1*, and *Vegfr3*. Treatment with SAR131675 significantly reduced the mRNA expression of these markers in the resiquimod-induced LN (Fig. 3B). In Western blot analysis, SAR131675 treatment decreased the resiquimod-induced increase of VEGFR-3 protein expression in the LN model (Fig. 3C). These data suggest that inhibition of VEGFR-3 by SAR131675 decreases lymphatic vessel expression in resiquimod-induced LN.

### Inhibition of VEGFR-3 decreases VEGF-C and VEGF-D expression in the resiquimod-induced lupus nephritis

**Fig. 4:**
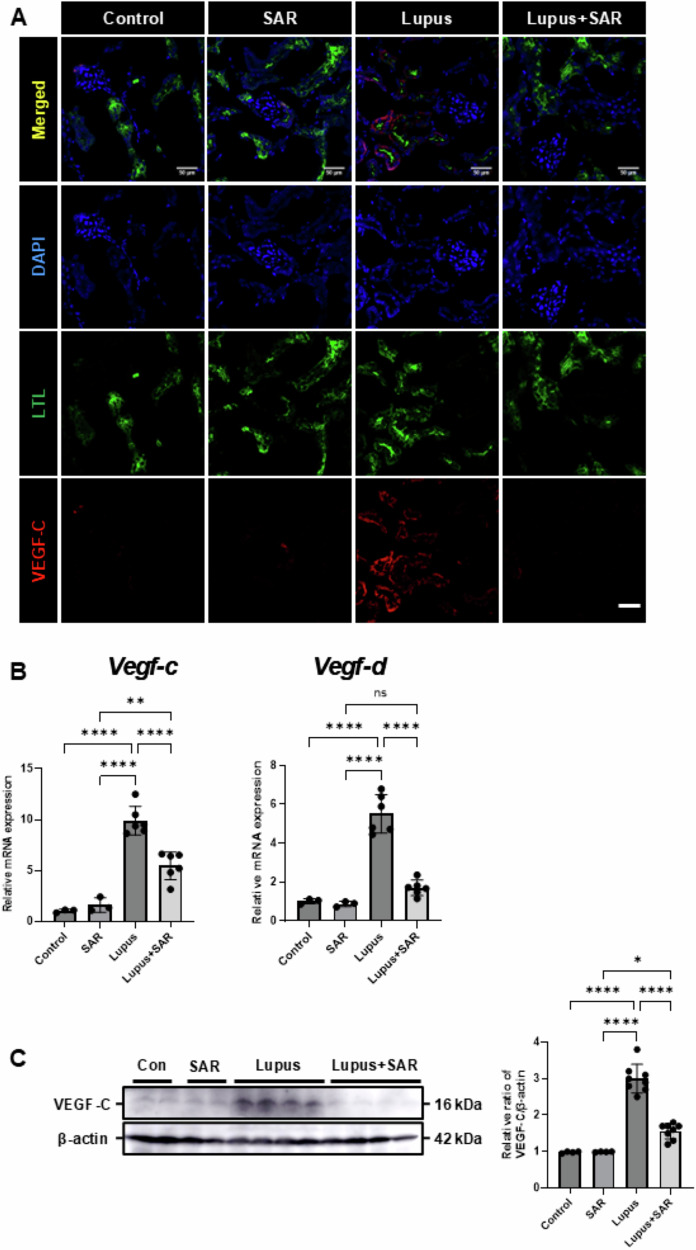
Treatment of SAR131675 decreases lymphangiogenic factor expression in the resiquimod-induced lupus nephritis. **A** Representative sections of kidneys from each experimental group of SAR131675 treatment in the resiquimod-induced LN were immunofluorescence stained with VEGF-C. *Lotus tetragonolobus* lectin (LTL) was used as a proximal tubule marker. Scale bar = 50 μm. **B** Quantitative RT-PCR analyses for *Vegf-c*, and *Vegf-d* mRNA expression were performed using mRNA from each experimental group of SAR131675 treatment in the resiquimod-induced LN. Expression levels were normalized to *Gapdh*. **C** Representative Western blot analysis of VEGF-C protein expression from each experimental group of SAR131675 treatment in the resiquimod-induced LN. The graph shows the densitometric quantification of Western blot bands by ImageJ software. Expression levels are normalized to β-actin. Statistical significance was determined using one-way ANOVA followed by Tukey’s post-hoc test. Data are expressed as mean ± SD. *P < 0.05, **P < 0.01, ****P < 0.0001 versus each group. Control, vehicle-treated group, SAR SAR131675-treated group, Lupus, resiquimod-treated group; Lupus+SAR, resiquimod and concomitantly SAR131675-treated group. DAPI 4’,6-diamidino-2-phenylindole, LN lupus nephritis, LTL *lotus tetragonolobus* lectin I, Vegf-c vascular endothelial growth factor-c, Vegf-d vascular endothelial growth factor-d.

To address the effect of VEGFR-3 inhibition by SAR131675 on lymphangiogenic factor expression in resiquimod-induced LN, we had a double immunofluorescence stain for VEGF-C and a proximal tubule marker, *Lotus tetragonolobus* lectin in the resiquimod-induced LN. In the resiquimod-induced LN, VEGF-C expression was significantly increased in the proximal tubules compared to the control or the SAR131675-only treated group. However, SAR131675 treatment significantly decreased VEGF-C expression in resiquimod-induced LN (Fig. 4A). In the resiquimod-induced LN model, we evaluated the expression of lymphangiogenic factors, specifically VEGF-C and VEGF-D. The induction of LN with resiquimod significantly increases the mRNA levels of *Vegf-c* and *Vegf-d*. SAR131675 treatment significantly reduced a resiquimod-induced increase of *Vegf-c* and *Vegf-d* mRNA expression (Fig. 4B). The VEGF-C protein level also increased in the resiquimod-induced LN. However, the SAR131675 treatment significantly decreased VEGF-C expression (Fig. 4C). These results suggest that inhibition of VEGFR-3 by SAR131675 decreases lymphangiogenic factor expression in resiquimod-induced LN.

### Inhibition of VEGFR-3 decreases tertiary lymphoid organ formation and inflammatory cytokine expression in the resiquimod-induced lupus nephritis

**Fig. 5:**
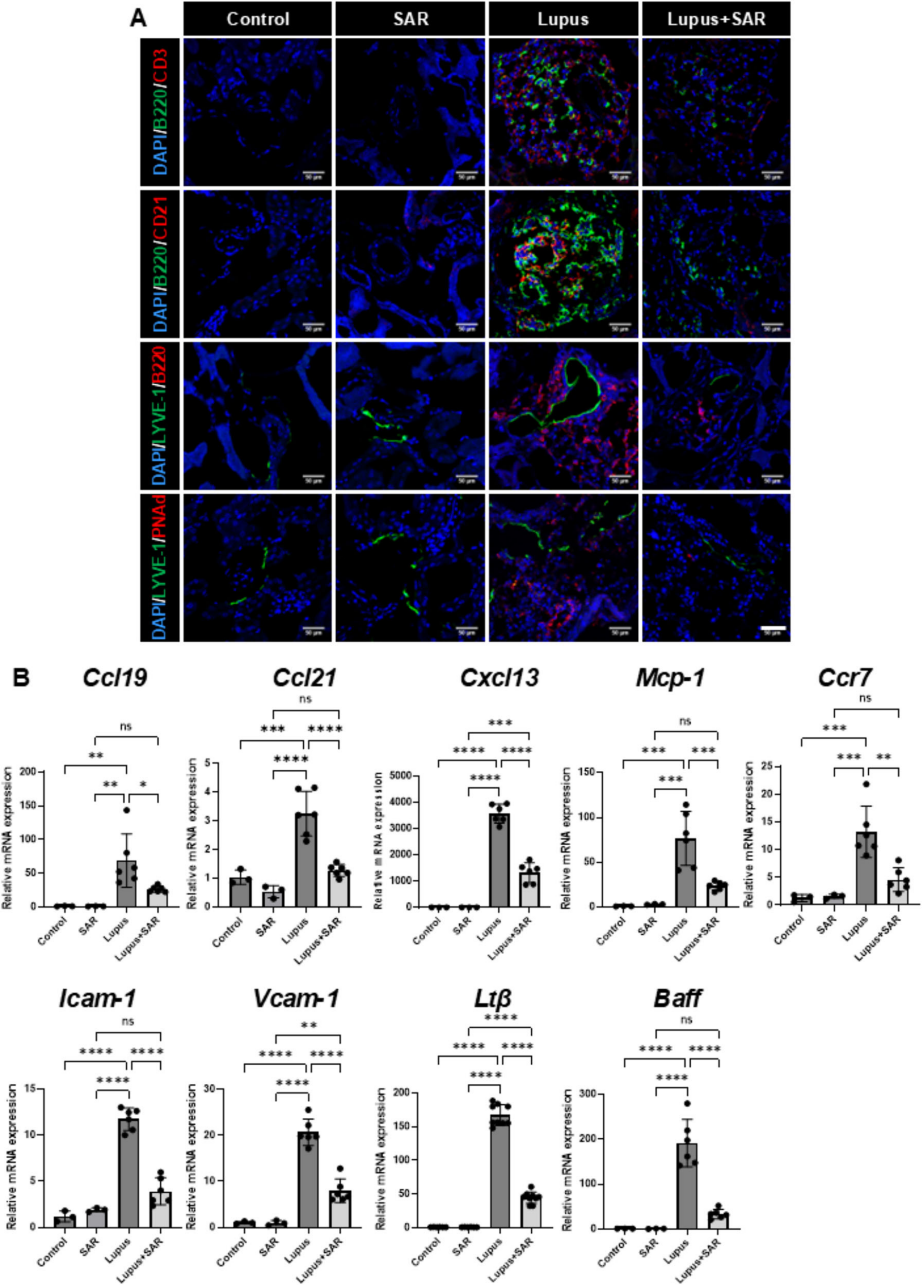
Treatment of SAR131675 decreases tertiary lymphoid organ formation and inflammatory cytokine expression in resiquimod-induced lupus nephritis. **A** Representative sections of kidneys from each experimental group of SAR131675 treatment in the resiquimod-induced LN were co-stained with B220/CD3, B220/CD21, LYVE-1/B220, LYVE-1/PNAd. TLOs formation is reduced, and renal function is improved after treatment with SAR131675. Scale bar = 50 μm. **B** Representative mRNA analysis of proinflammatory cytokines and chemokine expression. Expression levels are normalized to *Gapdh*. Statistical significance was determined using one-way ANOVA followed by Tukey’s post-hoc test. Data are expressed as mean ± SD. *P < 0.05, **P < 0.01, ***P < 0.001, ****P < 0.0001 versus each group. Control, vehicle-treated group; SAR, SAR131675-treated group; Lupus, resiquimod-treated group; Lupus+SAR, resiquimod and concomitantly SAR131675-treated group. DAPI 4’,6-diamidino-2-phenylindole, LN lupus nephritis, LYVE-1 lymphatic vessel endothelial hyaluronan receptor-1, PNAd peripheral node addressin, Ccl19 C-C motif chemokine ligand 19, Ccl21 C-C motif chemokine ligand 21, Cxcl13 C-X-C motif chemokine ligand 13, Mcp-1 monocyte chemoattractant protein-1, Ccr7 C-C chemokine receptor type 7, Icam-1 intercellular adhesion molecule-1, Vcam-1 vascular cell adhesion molecule 1, Ltβ lymphotoxin beta, Baff B-cell activating factor, *GAPDH* glyceraldehyde 3-phosphate dehydrogenase.

We observed severe tubulointerstitial inflammation with lymphoid aggregation in this resiquimod-induced LN model. These lymphoid aggregates are located around interlobar or arcuate arteries in the lupus model. We wondered what kinds of cells infiltrate these lymphoid aggregates and the relationship between lymphatic vessels and lymphoid aggregates. Therefore, we had immunofluorescence staining for B cells, T cells, follicular dendritic cells, lymphatic vessels, and high endothelial venules (HEVs). In Fig. 5A, there were lymphoid aggregates with CD3 (+) T cells, B220 (+) B cells, and CD21 (+) follicular dendritic cells (FDCs) in the resiquimod-induced LN model. We also observed LYVE-1 (+) lymphatic vessels with surrounding B220 (+) B cells and PNAd (+) HEVs. These studies suggest TLOs formation in resiquimod-induced LN. However, the inhibition of VEGFR-3 by SAR131675 decreased tubulointerstitial inflammation and lymphoid aggregation with decreased expression of LYVE1 (+) lymphatic vessels and PNAd (+) HEVs, suggesting a decrease in TLOs formation.

We also evaluate proinflammatory cytokines and chemokines related to forming TLOs. Proinflammatory cytokines and chemokines such as *Ccl19, Ccl21, Cxcl13, Mcp-1*, and *Ccr7* mRNA expressions were significantly increased in the resiquimod-induced LN model. However, treatment of SAR131675 decreased the resiquimod-induced increase of proinflammatory cytokines and chemokine expression. Cell adhesion molecules such as *Icam-1* and *Vcam-1* mRNA expression were significantly increased in the resiquimod-induced LN model. Treatment of SAR131675 significantly decreased the resiquimod-induced increase of cell adhesion molecule expression. There was also an increase in *Ltβ* and *Baff* mRNA expression in the resiquimod-induced LN. Treatment of SAR131675 significantly decreased *Ltβ* and *Baff* mRNA expression (Fig. 5B). These data suggest that inhibiting VEGFR-3 by SAR131675 decreases the resiquimod-induced TLOs formation in the murine LN model.

### Inhibition of VEGFR-3 regulates TLR7/MyD88/IFN-α signaling pathway in the resiquimod-induced lupus nephritis

**Fig. 6:**
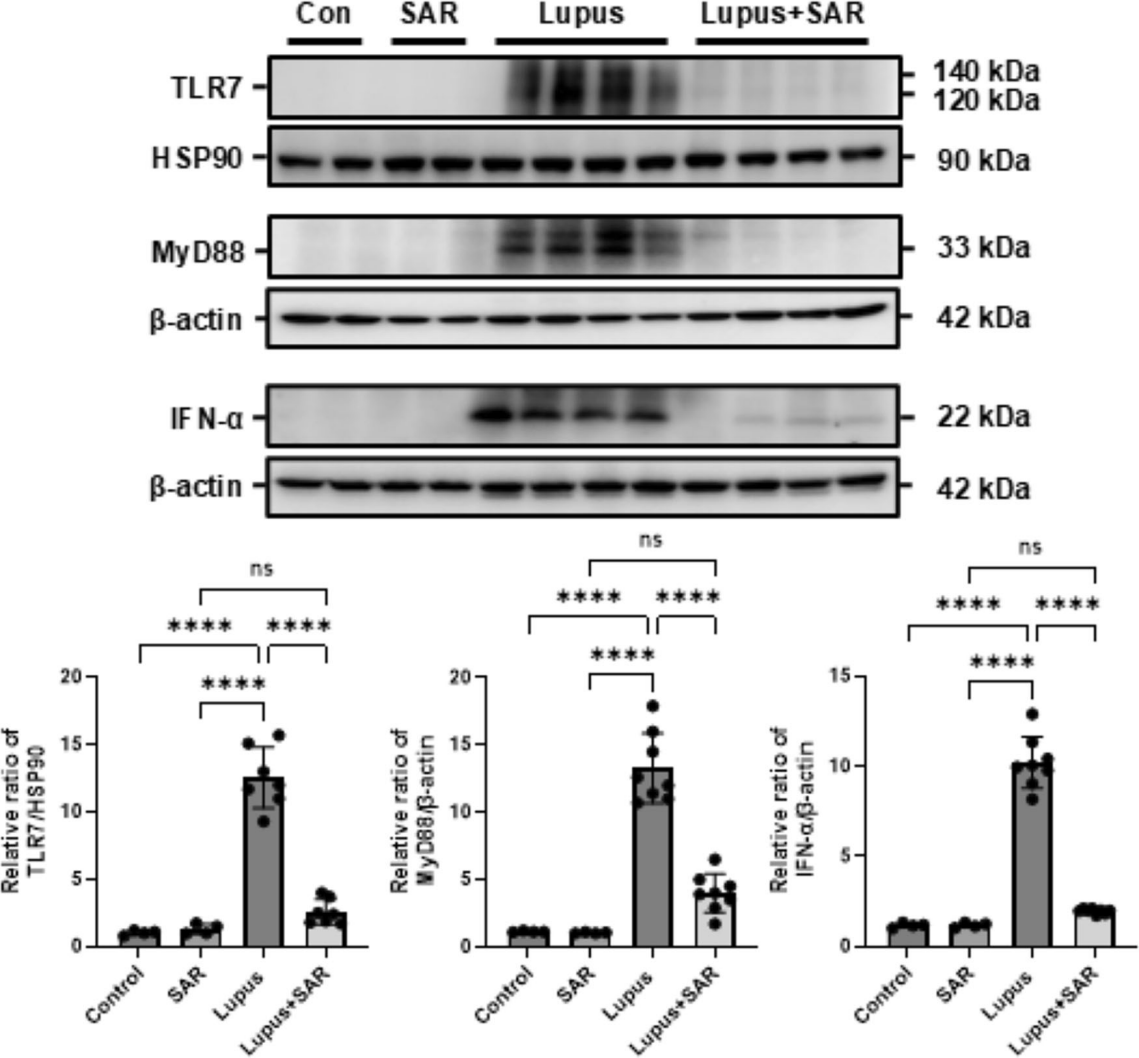
Treatment of SAR131675 decreases the resiquimod-induced increase of TLR7/MyD88/IFN-α signaling pathway in the resiquimod-induced lupus nephritis. Representative Western blot analysis of TLR7/MyD88/IFN-α protein expression from each experimental group of SAR131675 treatment in the resiquimod-induced LN. The graph shows the densitometric quantification of Western blot bands by ImageJ software. Expression levels are normalized to HSP90 or β-actin. Statistical significance was determined using one-way ANOVA followed by Tukey’s post-hoc test. Data are expressed as mean ± SD. ****P < 0.0001 versus each group. Control, vehicle-treated group; SAR, SAR131675-treated group; Lupus, resiquimod-treated group; Lupus+SAR, resiquimod and concomitantly SAR131675-treated group. HSP90 heat shock protein 90, IFN-α interferon-α, LN lupus nephritis, MyD88 myeloid differentiation primary response 88, TLR7 toll-like receptor 7.

Type I interferon (IFN) response is associated with many autoimmune diseases such as rheumatoid arthritis (RA), SLE, Sjogren’s syndrome, and systemic sclerosis with driving an inflammatory response. To address the effect of VEGFR-3 inhibition by SAR131675 on TLR7/MyD88/IFN-α signaling pathway, we evaluated toll-like receptor 7 (TLR7), myeloid differentiation primary response 88 (MyD88), and IFN-α expression in the resiquimod-induced LN. TLR7, MyD88, and IFN-α expression were significantly increased in the resiquimod-induced LN model. However, SAR131675 treatment decreased the resiquimod-induced increase of TLR7, MyD88, and IFN-α expression (Fig. 6). These data suggest that inhibiting VEGFR-3 by SAR131675 significantly decreases the resiquimod-induced increase of the type I IFN signaling pathway.

## Discussion

The kidney lymphatic systems play important roles in interstitial fluid homeostasis and tuning immune responses, such as reabsorption of cytokines, growth factors, and immune cells [29]. Pathologically, inflammation can trigger lymphangiogenesis and initiate a cycle of immune response and fibrosis within the kidneys [29, 30]. Targeting the lymphatic system could be a novel kidney disease therapeutics. This study examined the effect of VEGFR-3 inhibition by SAR131675 in the murine LN model. We show that VEGFR-3 inhibition improves glomerular and tubulointerstitial inflammation and decreases inflammation-induced lymphangiogenesis and TLOs formation in resiquimod-induced LN. This protective effect may regulate the TLR7/MyD88/IFN-α signaling pathway.

LN occurs in half of patients with SLE, causes progressive renal failure, and is associated with a high mortality rate compared with those without nephritis [24]. Proliferative forms of LN (class III, IV, or III/IV + V) are the highest risk for requiring kidney replacement therapy (KRT) [24]. In addition, tubulointerstitial lesions, including interstitial inflammatory cell infiltration, interstitial fibrosis, and tubular atrophy, are significant predictors of renal outcomes in patients with LN [19, 31]. Therefore, targeting tubulointerstitial lesions, such as regulating interstitial inflammation and fibrosis, could significantly improve treating LN. Local autoimmunities, such as immune complex formation, infiltrating cells of hematological origin, or kidney resident cells, are important in the pathogenesis of LN [20].

In our previous work, VEGF-C and VEGF-D are involved in renal fibrosis-induced lymphangiogenesis in the unilateral ureteral obstruction (UUO) model [32]. Among them, macrophage and proximal tubule cells express VEGF-C and induce UUO-induced lymphangiogenesis. Our current study shows an increase in lymphangiogenic factors such as *Lyve-1*, *Pdpn*, *Prox-1*, and *Vegfr3* mRNA in our lupus model. The VEGF-C and VEGFR-3 also increase expression with LTL (+) proximal tubule cells and LYVE-1 (+) lymphatic vessels, respectively. SAR131675 treatment decreases the LN-induced increase of VEGF-C and VEGFR-3 in the LTL (+) proximal tubule cells and LYVE-1 (+) lymphatic vessels. Hwang et al. reported that inhibiting VEGFR-3 signaling by SAR131675 decreases diabetic nephropathy by regulating lymphatic vessel proliferation [33]. Nihei et al. reported that treatment with the VEGFR inhibitor axitinib or the VEGFR-3 inhibitor SAR131675 impaired inflammation-induced lymphangiogenesis and improved oxygen saturation in the murine aspiration model [34]. Therefore, inhibiting VEGFR-3 signaling regulates inflammatory response and lymphangiogenesis, resulting in improved organ functions.

In the tubulointerstitial lesion in LN, the role of lymphoid aggregates with T cells and B cells in LN remains to be fully appreciated. TLOs formation relies on inducible inflammatory triggers, such as CXCL13 and CCL9, as well as lymphotoxin αβ [35]. In our lupus model, TLOs comprise T cells, B cells, and CD21 (+) FDCs with lymphatic vessels and PNAd (+) HEVs close to the renal pelvis and large vascular structures. Dorraji et al. reported a high correlation between TLOs and anti-dsDNA antibody titer in the LN model [36]. The initiation of an inflammatory process in the tubulointerstitial area may occur by activating tubular cells and/or resident monocyte/dendritic cells [37]. The inflammatory responses promote the formation of autoantigens, the expression of lymphotoxin-α1β2, and interaction with the lymphotoxin-β receptor, which leads to the formation of organized TLOs [37]. The release of lymphoid chemokines favors the development of T cell accumulation, and further recruitment of T and B lymphocytes might be supported by the expression of HEVs that allow recruitment of naïve lymphocytes [37]. B cells are prominent cell populations in TLOs, which comprise active germinal centers (GCs), indicating the proliferation and differentiation of reactive B cells [38]. Local stromal cells, such as fibroblastic reticular cells (FRCs) and FDCs, play a role in TLOs formation, which produces homeostatic chemokines, such as CXCL13, CCL21, and CCL19, and forms a network for B cell migration [35, 39]. In our lupus model, increasing proinflammatory cytokines such as *Cxcl13*, *Ccl19, Ltβ*, and *Baff* mRNA levels trigger the TLOs formation. After the inhibition of VEGFR-3 by SAR131675, lupus-induced tubulointerstitial inflammation and TLOs formation were attenuated through the downregulation of proinflammatory cytokines, lymphangiogenic factors, VEGF-C and -D, and VEGFR-3. Our model also shows that LYVE-1 (+) lymphatic vessels and PNAd (+) HEVs surround the TLOs, indicating active lymphangiogenesis and immune cell trafficking. Therefore, TLOs are essential in the induction of local inflammatory responses, such as tubulointerstitial inflammation in LN, and contribute to disease progression.

Advances in understanding the molecular mechanisms of innate immunity, with elucidation of the endosomal TLRs, have suggested that type I IFN, particularly IFN-α, is a central mediator in the pathogenesis of SLE [40]. Persistent type I IFN stimulation increases T and B cell activity, produces autoantibodies, and induces autoimmune diseases [41]. Recently, TLR-7 and TLR-9 have contributed to the development of autoimmune diseases such as rheumatoid arthritis, SLE, and psoriasis [26]. In our LN model, we applied 100 μg of resiquimod (R848), a TLR-7 agonist, topically every other day. This protocol has successfully induced a lupus-like model with renal dysfunction, albuminuria, and immune complex-mediated glomerulonephritis. Our LN model also shows severe tubulointerstitial inflammation with lymphoid cell aggregations and an increase of LYVE-1 (+) and VEGFR-3 (+) lymphatic vessel expression. Therefore, we have questions about the effects of inhibition of lymphatic vessel expression by regulating VEGF-C/VEGFR-3 signaling pathway. Our study demonstrates that VEGFR-3 inhibition reduces TLR7/MyD88/IFN-α signaling in lupus nephritis. While the exact mechanism remains unclear, it is possible that VEGFR-3 inhibition reduces lymphangiogenesis, subsequently affecting antigen presentation and immune cell trafficking, thereby dampening TLR7-mediated inflammation activation. Further studies are required to elucidate the direct or indirect interactions between VEGFR-3 and TLR7/MyD88/IFN-α signaling.

SAR131675 is known as a potent and selective inhibitor of VEGFR-3. However, it was moderately active on VEGFR-2. Alam et al. reported that SAR131675 has anti-lymphangiogenic, antitumoral, and antimetastatic activities regulating tumor-associated macrophage (TAM) infiltration [42]. VEGFR-3 can form heterodimers with VEGFR-2, which proteolytically processed forms of VEGF-C and VEGF-D [5]. Therefore, SAR131675 might have off-target effects on the regulation of renal inflammation and lymphangiogenesis. Moreover, human pharmacokinetic and dosing data for SAR131675—either in lupus nephritis or other indications—remain limited.

Our study has several limitations: There is no direct interaction between VEGFR-3 and TLR7 activation, unproven in situ autoantibody production by TLO-resident B cells, reliance on a single animal model for lupus nephritis, short-term treatment and lack of functional lymphatic readouts and a small number of the human cohort (*n* = 22) for evaluation the association of lymphatic vessel expression with histopathological activity and serologic markers in human lupus nephritis. Future studies should address these limitations by isolating viable TLO-resident B cells and single-cell RNA sequencing and using other murine lupus models or alternative modes of VEGFR-3 targeting for further study. A larger-scale human cohort study might have provided a mechanistic link between histopathologic activity and lymphatic vessel expression in lupus nephritis.

Collectively, our data highlights the therapeutic potential of targeting lymphatic proliferation by VEGFR-3 inhibition in LN. Modulation of the lymphatic network may provide a novel approach to treating chronic inflammation and attenuating renal autoimmune response. Future studies must elucidate the precise mechanisms by which lymphangiogenesis contributes to proliferative glomerular and tubulointerstitial lesions in LN.

## Materials and methods

### Analysis of human kidney biopsy

For analysis of lymphatic vessel expression in LN, human kidney tissues were obtained from renal biopsy specimens, which were pathologically diagnosed with LN according to the Revised International Society of Nephrology/Renal Pathology Society (ISN/RPS) classification for lupus nephritis (class I to V, *n* = 22) [25]. This study was reviewed and approved by the Institutional Review Board of Jeonbuk National University Hospital (CUH-2020-07-032-004). Written informed consent for publication was obtained from the patients. We reviewed medical records and analyzed their clinical characteristics (Table 1). Immunohistochemical staining for lymphatic vessels was performed by using D2-40 mouse monoclonal antibody (cat#05463645001, prediluted, Roche Diagnostics, Rotkreuz, Switzerland) on BenchMark ULTRA system (Roche Diagnostic), and D2-40 positive lymphatic vessels were counted in magnification of 200×. We analyzed the relationship between the expression of D2-40-positive lymphatic vessels and the modified NIH activity indexes and serum anti-dsDNA antibody levels.

### Animal experiment

The Institutional Animal Care and Use Committee of Jeonbuk National University Hospital reviewed and approved the experimental animal protocol (cuh-IACUC-2018-18). The number of animals per group was minimized while maintaining statistical reliability, in accordance with the 3R principles (Replacement, Reduction, Refinement) and our institutional ethical guidelines. Male Balb/c mice (7–8-week-old) were purchased from Orient Bio Inc. (Seongnam, Korea) and maintained in the animal room with controlled temperature (23 ± 1 °C), humidity (40-60%), light source (12–12 h light-dark cycle), with food and water access *ad libitum*. For animal experiments, mice were randomly assigned to one of four groups using a computer-generated randomization sequence: control (Con), SAR (SAR131675 treatment), LN (Lupus), and LN with treatment of SAR131675 (Lupus+SAR) (*n* = 6/group). The allocation was performed by an investigator blinded to the study design to minimize bias. For induction of a lupus-like model, the dorsal skin of mice was shaved and given topical treatment every other day with 100 μg resiquimod (R848; ALX-420-038; Enzo Life Sciences, NY, USA) dissolved in 100 μL acetone during the 8-week treatment [26]. SAR131675 (S2842, Selleck Chemicals, Houston, TX), the VEGFR-3 inhibitor, was suspended in 100 μL 5% dimethyl sulfoxide (DMSO) and dissolved in 900 μL corn oil (Sigmal Chemical Co.). SAR131675 (100 mg/kg) was administered concomitantly daily through oral gavage. Corn oil was used as a vehicle. Exclusion criteria were pre-established before the experiment. Mice were excluded from the analysis if they have severe illness unrelated to the experimental conditions, unexpected mortality before study endpoints, or failure to develop lupus-like symptoms in the LN group. One mouse was excluded because of unexpected mortality before study endpoints in the LN group.

After an 8-week protocol, mice were anesthetized via intraperitoneal injection of a cocktail mixture of ketamine (100 mg/kg, Yuhan, Seoul, Korea) and xylazine (10 mg/kg, Bayer Korea, Seoul, Korea). An 800 µl whole blood sample was obtained by intracardiac puncture. The kidneys were harvested, prepared for histological examination, snap-frozen in liquid nitrogen, and stored at -80 °C for further studies. The spleen was obtained and weighed.

### Measurement of renal function

The mice were placed in metabolic cages to collect 24 h urine samples, and we measured urine albumin and creatinine excretion using the Albuwell M kit (Ethos Bioscience, Logan Township, NJ, USA) and the Creatinine Companion (Ethos Bioscience) [43]. Urinary albumin excretion was expressed as the ratio of urinary albumin to creatinine (μg/mg). Serum creatinine was measured by an enzymatic method using an automatic analyzer (Hitachi7180, Tokyo, Japan).

### Measurement of serum anti-dsDNA antibody levels

Serum anti-dsDNA antibody levels were measured by enzyme-linked immunosorbent assay (ELISA) kit (LBIS mouse anti-dsDNA antibody ELISA kit, cat#637-02691, FUJIFILM Wako Chemicals, Osaka, Japan) according to the manufacturer’s instructions.

### Renal histology

The kidneys were fixed in 4% paraformaldehyde and embedded in paraffin. The paraffin block was prepared into 5 μm sections and stained with Hematoxylin and Eosin (H&E). Slides were examined with a Carl Zeiss Z1 microscope (Carl Zeiss, Oberkochen, Germany). The severity of glomerular inflammation, mesangial cell proliferation, crescent formation, necrosis, tubulointerstitial changes, and vascular injury was graded semi-quantitatively: 0, no change; 1, mild; 2, moderate; and 3, severe. Kidney injury score represents the sum of individual scores in ten randomly chosen, non-overlapping fields at a magnification of 200× [44]. The scores for the crescent formation and necrosis were doubled to reflect the severity of the lesions. The maximum score was 24.

For immunofluorescence staining, freshly frozen kidney tissues were fixed with 4% paraformaldehyde at 4 °C for 24 h, dehydrated with 20% sucrose for 24 h, and embedded in Tissue Plus OCT cryostat compound (Fisher Scientific, Pittsburgh, USA). Cryosections were prepared at 6-µm thickness on the ProbeOn Plus microscope slide (Fisher Scientific, Pittsburgh, USA). The tissue samples were permeabilized for 10 min with 1% Triton X-100 and then followed by an incubation step with blocking buffer (Protein Block Serum-Free; DAKO, Agilent Technologies, CA, USA) at room temperature. The tissue samples were incubated with a FITC-labeled *Lotus tetragonolobus* lectin (Vector Laboratories, Burlingame, CA, USA), a rat anti-mouse B220 (550286; dilution 1:100; BD pharmingen), a rabbit anti-CD3 (ab16669; dilution 1:100; Abcam), a rabbit anti-mouse LYVE-1 (11-034; dilution 1:100; AngioBio Co), a rabbit anti-mouse CD21 (ab75985, dilution 1:100; Abcam), a rabbit anti-VEGF-C (PA5-29772; dilution 1:200; Invitrogen), a gout anti-mouse VEGFR-3 (AF743; dilution 1:100; R&D systems), a rabbit anti-mouse CD21 (ab75985; dilution 1:250; Abcam), a rat anti-mouse PNAd (553863; dilution 1:100; BD pharmingen), a rabbit anti-mouse IgG(H + L) FITC-conjugated (A90-117F; dilution 1:100; Bethyl Laboratories), a goat anti-mouse IgM FITC-conjugated (A90-201F; dilution 1:100; Bethyl Laboratories), a rat anti-mouse C3 (ab11862; dilution 1:100; Abcam) antibodies. The next day, slides were exposed to a Cy3-labeled/FITC-labeled secondary antibody (1:1,000; Thermo Fisher Scientific, MA, USA) for 1 h at room temperature in the dark. Nuclear staining was operated using 4’,6-diamidino-2-phenylindole (DAPI, Invitrogen, Waltham, MA, USA) for 1 min lucifugal at room temperature. Slides were examined with a Carl Zeiss LSM 880 super-resolution confocal laser scanning microscope (Carl Zeiss, Oberkochen, Germany). All images were analyzed through the ImageJ software program (http://rsb.info.nih.gov/ij).

### Western blotting

The Western blot analysis was assessed as described previously [45]. Kidney tissues lysates were separated by 8–12% SDS-PAGE gel and wet-transfer onto PVDF membrane (Bio-Rad Laboratories, Hercules, CA, USA) and followed by blocking with 5% non-fat milk solution (Becton, Dickinson & Company, Sparks, MD, USA) for 1 h at room temperature; membranes were then incubated overnight with a rabbit anti-VEGF-C (ab9546, dilution 1:1000; Abcam), goat anti-VEGFR-3 (AF743, dilution 1:1000; R&D Systems), rabbit anti-TLR7 (#82658, dilution 1:1,000; Cell Signaling Technology), rabbit anti-MyD88 (#4283, dilution 1:1000; Cell Signaling Technology), rabbit anti-IFN-α (#PA5-115430, dilution 1:1000; Thermo Fisher Scientific), mouse anti-β-actin (sc-47778, 1:2000; Santa Cruz Biotechnology, Inc.), rabbit anti-glyceraldehyde 3-phosphate dehydrogenase (GAPDH; AP0063, dilution, 1:2000; Bioworld Technology, Inc., Danvers, MA, USA), rabbit anti-heat shock protein 90 (HSP90) polyclonal antibody (ADI-SPA-836, dilution 1:1000; Enzo Biochem Inc, Farmingdale, NY, USA) at 4 °C. Membranes were incubated with conjugated secondary antibodies for 1 h at room temperature. Protein expression was detected with a chemiluminescent detection kit according to the manufacturer’s protocols (Immobilon® Western Chemiluminescent HRP Substrate, Merck KGaA, Darmstadt, Germany), and figures were captured by a densitometric scanner (ImageQuant LAS 4000 Mini, GE Healthcare Life Sciences, Piscataway Township, NJ, USA). The GAPDH, HSP90, and β-actin were applied as loading control.

### RNA isolation and quantitative PCR analyses

Isolation of RNA and quantitative real-time reverse transcription polymerase chain reaction (qRT-PCR) was assessed as previously described [45]. In brief, frozen kidney sections were homogenized, and TRIzol reagent (Invitrogen, Carlsbad, CA, USA) was used to isolate total RNA. A Transcriptor First Stand cDNA Synthesis Kit (Roche Diagnostic, Mannheim, Germany) was assessed according to the manufacturer’s instructions for synthesizing cDNA from total RNA. qRT-PCR of mouse *Lyve-1, Vegfr3, Vegf-c, Vegf-d, podoplanin, Prox-1, Ccl19, Ccl21, Cxcl13, Mcp-1, Ccr7, Icam-1, Vcam-1, Ltβ*, and *Baff* mRNA expression were assessed in a Rotor-Gene Q (Qiagen, Germantown, MD, USA). Primers used for each gene (Table 2) were designed using Primer-BLAST (NCBI, www.ncbi.nlm.nih.gov/tools/primer-blast/). Each cDNA transcript was diluted 10-fold and amplified in a 10 µl volume with SYBR Green PCR Master Mix (Applied Biosystems, Inc., Carlsbad, CA, USA). The PCR program was denaturation at 95 °C for 10 min followed by 40 amplification cycles of 95 °C for 10 s and 60 °C for 30 s. *Gapdh* mRNA expression was examined in every sample for equalization of each reaction.

**Table 2:** Primer list for target genes used for qRT-RCR in this study.

Gene	Accession no.	Forward primer (5’ – 3’)	Reverse primer (5’ – 3’)
*Gapdh*	NM_008084	TCATCAACGGGAAGCCCATC	AGACACCAGTAGACTCCACGA
*Lyve-1*	NM_053247	CCGACACCTGGGTTAACTCC	TGGTGGCAGAAACAGGTGTT
*Vegfr3*	NM_008029	AGTCGGAGCCTTCTGAGGAT	CCTCCTCTAGGTTGGCCTCT
*Pdpn*	NM_010329	CAGGTACAGGAGACGGCATG	GGTTGCTGAGGTGGACAGTT
*Prox-1*	NM_008937	TCGCAGCTCATCAAGTGGTT	TAGTGCATGTTGAGGGCTCG
*Vegf-c*	NM_009506	CAGCACAGGTTACCTCAGCA	TTAGCTGCCTGACACTGTGG
*Vegf-d*	NM_010216	CTGCTCGGATCTGTTGTTCA	GTGTACTTGGTGCAGGGCTT
*Icam-1*	NM_010493	TTTTGGAGCTAGCGGACCAG	CCGCTCAGAAGAACCACCTT
*Vcam-1*	NM_011693	GCCAAATCCACGCTTGTGTT	AGGTCTCCCATGCACAAGTG
*Mcp-1*	NM_011333	CAGCCAGATGCAGTTAACGC	CCTCTTGAGGGCTGTGTCTG
*Ccl19*	NM_011888	TGGGAACATCGTGAAAGCCT	TCTGGTGCTGTTGCCTTTGT
*Ccl21*	NM_011124	TGTGCAAACCCTGAGGAAGG	CCTCTTGAGGGCTGTGTCTG
*Ccr7*	NM_001301713	TTGACCGCTACGTAGCCATC	AGTGAGCATCTCAGCGTGTC
*Ltβ*	NM_008518	TACTTCCCTGGTGACCCTGT	TGAGAGGCTAGAGGGTGAGG
*Cxcl13*	NM_018866	CTCTCCAGGCCACGGTATTC	TTGGCACGAGGATTCACACA
*Baff*	NM_033622	CGACACGCCGACTATACGAA	GCAAAGATGGGGTCCGTGTA

### Statistical analysis

The data are expressed as the mean ± standard deviation (SD). We used the Shapiro-Wilk test to confirm whether the data set was normally distributed. A one-way analysis of variance (ANOVA) was used for normally distributed data to evaluate differences within groups, followed by an individual comparison between groups with the Tukey post hoc test. Spearman’s rank correlation coefficient and Kendall’s tau correlation coefficient test were used to analyze lymphatic vessel expression and the modified NIH activity indexes or serum anti-dsDNA antibody levels. We used GraphPad Prism 10.2 or SPSS statistics version 27 to statistically analyze and conduct graphs; a P-value < 0.05 was considered statistically significant.

## Supplementary information

Western blot original

## Data Availability

Original data from this study will be available from the corresponding author upon request.
