# Supplementary material for: Inhibition of VEGFR-3 by SAR131675 decreases renal inflammation and lymphangiogenesis in the murine lupus nephritis model
Source: Cell Death Discov. 2025 Jul 12;11:320. doi: 10.1038/s41420-025-02624-4 (PMC12255756; doi:10.1038/s41420-025-02624-4)
Supplement: Supplementary file 1 — Western blot original [file 41420_2025_2624_MOESM1_ESM.pptx]

## Slide 1
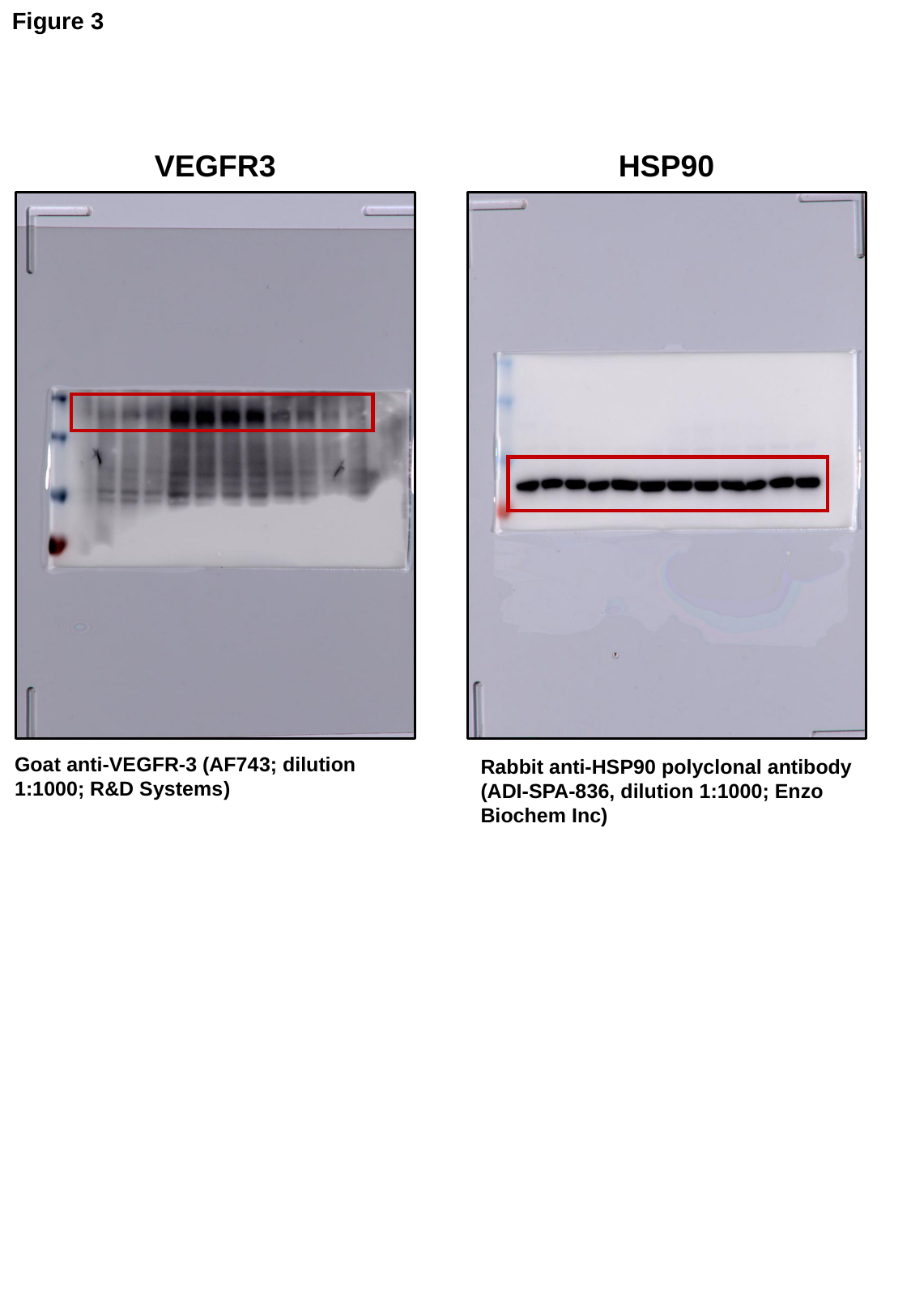

Figure 3
VEGFR3
HSP90
Goat anti-VEGFR-3 (AF743; dilution 1:1000; R&D Systems)
Rabbit anti-HSP90 polyclonal antibody (ADI-SPA-836, dilution 1:1000; Enzo Biochem Inc)

## Slide 2
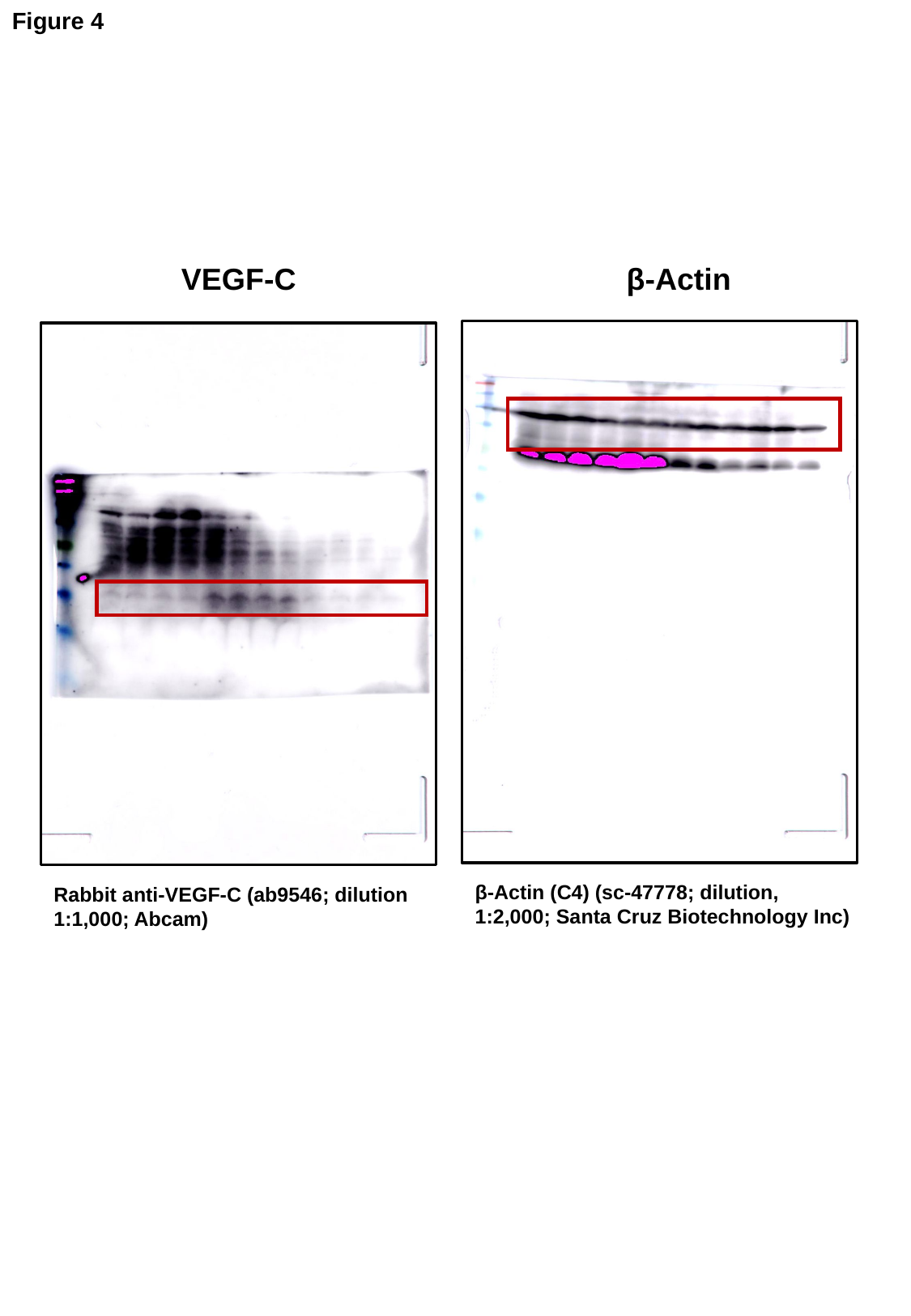

Figure 4
VEGF-C
β-Actin
β-Actin (C4) (sc-47778; dilution, 1:2,000; Santa Cruz Biotechnology Inc)
Rabbit anti-VEGF-C (ab9546; dilution 1:1,000; Abcam)

## Slide 3
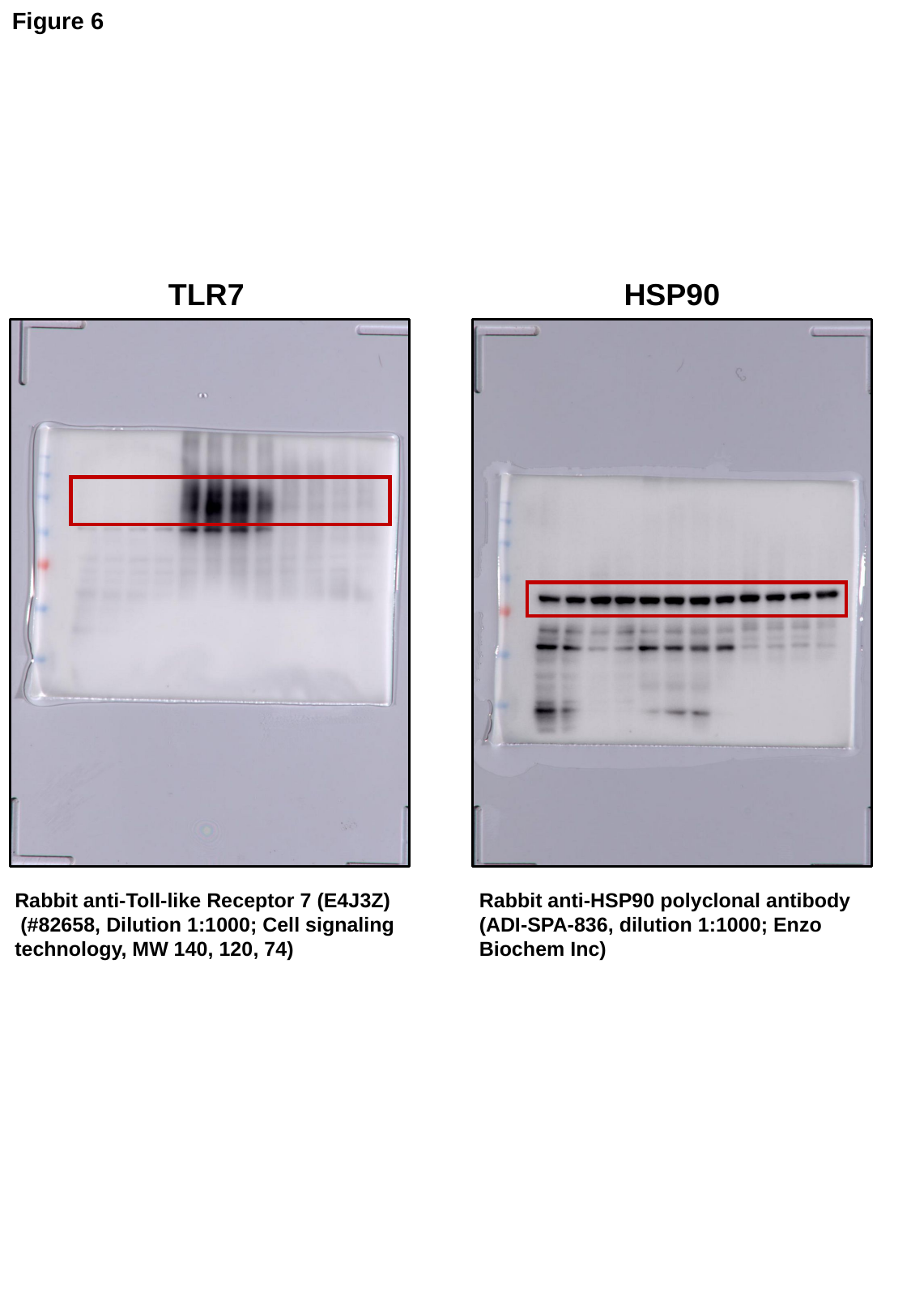

Figure 6
HSP90
TLR7
Rabbit anti-HSP90 polyclonal antibody (ADI-SPA-836, dilution 1:1000; Enzo Biochem Inc)
Rabbit anti-Toll-like Receptor 7 (E4J3Z)
 (#82658, Dilution 1:1000; Cell signaling technology, MW 140, 120, 74)

## Slide 4
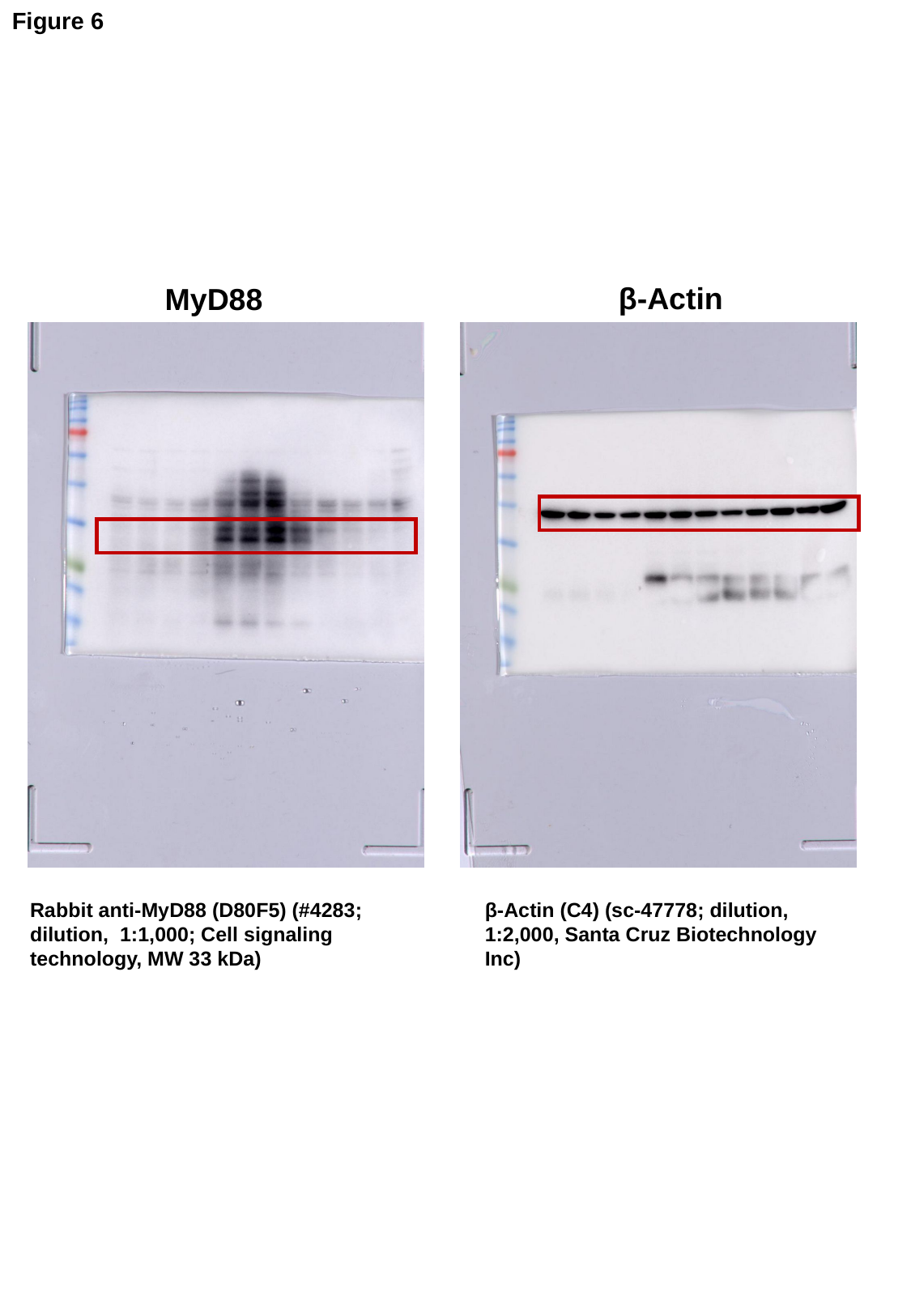

Figure 6
β-Actin
MyD88
Rabbit anti-MyD88 (D80F5) (#4283; dilution, 1:1,000; Cell signaling technology, MW 33 kDa)
β-Actin (C4) (sc-47778; dilution, 1:2,000, Santa Cruz Biotechnology Inc)

## Slide 5
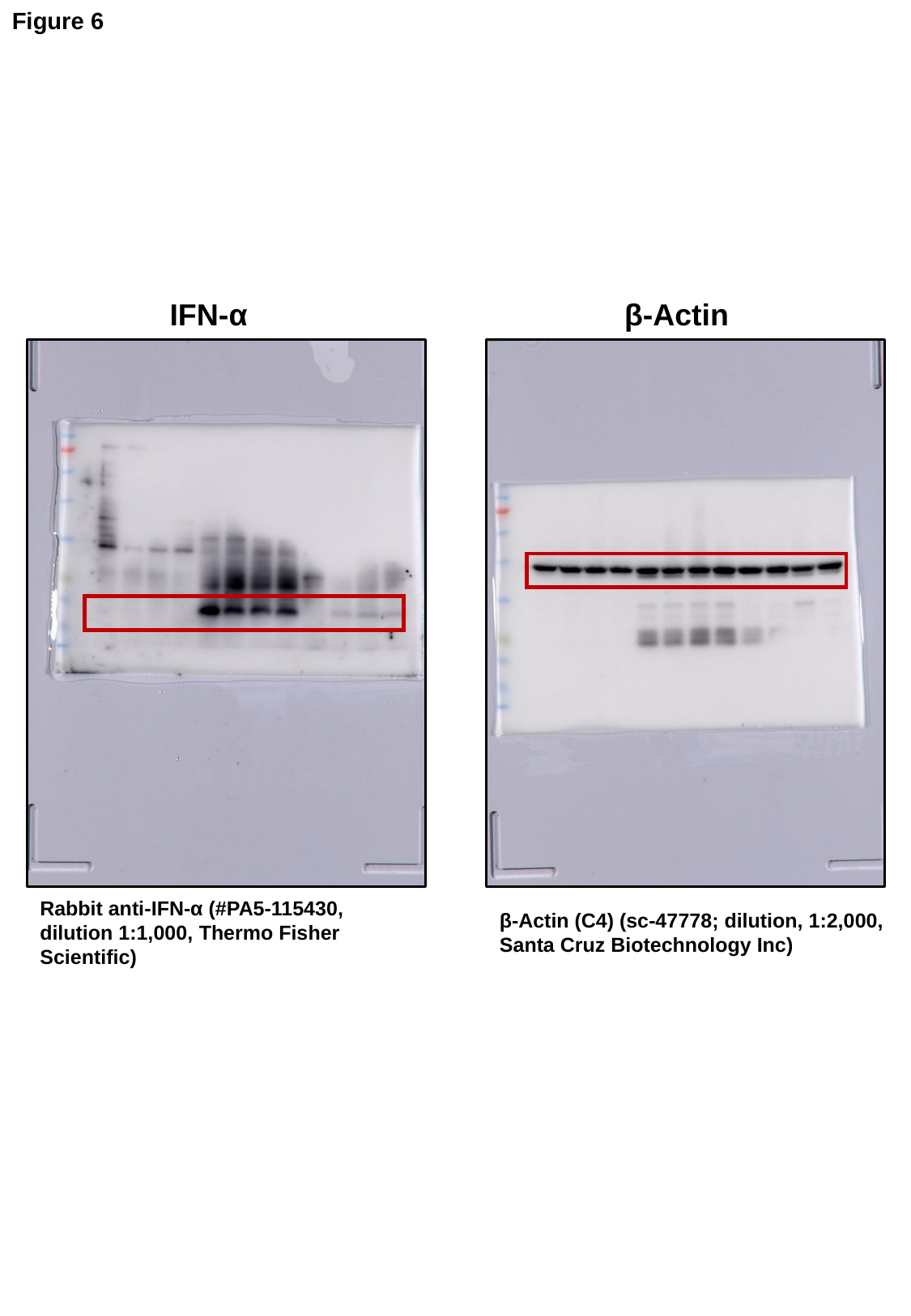

Figure 6
IFN-α
β-Actin
Rabbit anti-IFN-α (#PA5-115430, dilution 1:1,000, Thermo Fisher Scientific)
β-Actin (C4) (sc-47778; dilution, 1:2,000, Santa Cruz Biotechnology Inc)
